# Effect of Elevated Temperature Annealing on Nafion/SiO_2_ Composite Membranes for the All-Vanadium Redox Flow Battery

**DOI:** 10.3390/polym10050473

**Published:** 2018-04-26

**Authors:** Sixiu Zeng, Liuli Zeng, Rui Wang, Wei Guo, Haolin Tang

**Affiliations:** State Key Laboratory of Advanced Technology for Materials Synthesis and Processing, Wuhan University of Technology, Wuhan 430070, China; zengsixiu@whut.edu.cn (S.Z.); 18086458885@163.com (L.Z.); 512651259@whut.edu.cn (R.W.)

**Keywords:** Nafion/SiO_2_ membrane, elevated temperature annealing, all-vanadium redox flow battery

## Abstract

Conducting Nafion/SiO_2_ composite membranes were successfully prepared using a simple electrostatic self-assembly method, followed by annealing at elevated temperatures of 240, 270, and 300 °C. Membrane performance was then investigated in vanadium redox flow batteries (VRB). These annealed composite membranes demonstrated lower vanadium permeability and a better selectivity coefficient than pure Nafion membranes. The annealing temperature of 270 °C created the highest proton conductivity in the Nafion/SiO_2_ composite membranes. The microstructures of these membranes were analyzed using transmission electron microscopy, small-angle X-ray scattering, and positron annihilation lifetime spectroscopy. This study revealed that exposure to high temperatures resulted in an increase in the free volumes of the composite membranes, resulting in improved mechanical and chemical behavior, with the single cell system containing composite membranes performing better than systems containing pure Nafion membranes.

## 1. Introduction

The growth in global consumption of fossil fuels, such as coal, natural gas, and oil, will eventually lead to the exhaustion of these finite resources. Therefore, finding alternative and renewable energy sources is necessary [1,2]. Electrochemical storage systems are a potential solution for many energy storage applications because of their flexibility in terms of localization, efficiency, and scalability [3]. All-vanadium redox flow batteries (VRB) have received widespread attention given their potential as clean and efficient power devices for large-scale energy storage and residential power sources, due to their high energy efficiency and capacity, flexible design, long cycle life, low operating costs, and fast response times [4,5,6]. VRB use porous carbon felt electrodes and ion exchange membranes in the presence of two electrolyte tanks containing V(IV)/V(V) and V(II)/V(III) as electrolytes in sulfuric acid solution as negative and positive electrolytes, respectively [3,7,8,9]. Ion exchange membranes play an important role in VRB, whose function is to effectively separate positive and negative electrolytes, preventing vanadium ion crossover, and creating efficient hydrogen ion flow to complete a current loop. Perfluorosulfonic acid (PFSA) membranes, such as the Nafion membranes produced by DuPont, are widely used in vanadium redox flow batteries because of their good chemical, mechanical, and thermal stability, as well as their relatively high levels of proton conductivity when present in a hydrated state [10]. Although this membrane has many advantages as an ion exchange membrane, this battery design suffers from a dramatic decline in columbic efficiency and an increase in self-discharge events due to uncontrolled vanadium ion crossover [11,12,13]. Therefore, finding methods to reduce vanadium ion crossover and improve ion selectivity are important issues when developing durable ion exchange membranes (IEMs) for VRB.

Efforts have been directed toward the development of IEMs that exhibit low vanadium ion permeability [7,14,15,16,17,18,19], including many solution casting or sol-gel methods designed to modify Nafion membranes by introducing vanadium blockers into their matrices to reduce vanadium ion crossover [20]. The vanadium ion permeability of these composite membranes remarkably decreased compared with unmodified Nafion membranes, resulting in improved performance. However, the introduction of inorganic dopants may decrease the conductivity of the membrane, especially if the inorganic dopants were not introduced using an orderly process. To solve this problem, heat-treatment of these composite membranes resulted in improved distribution of inorganic dopants [21,22]. However, the proton conductivities of many recast Nafion membranes decrease as the heat treatment temperature increases, meaning that the overall performance of many of these modified membrane systems was no better than the parent Nafion212 [11].

**Figure 1:**
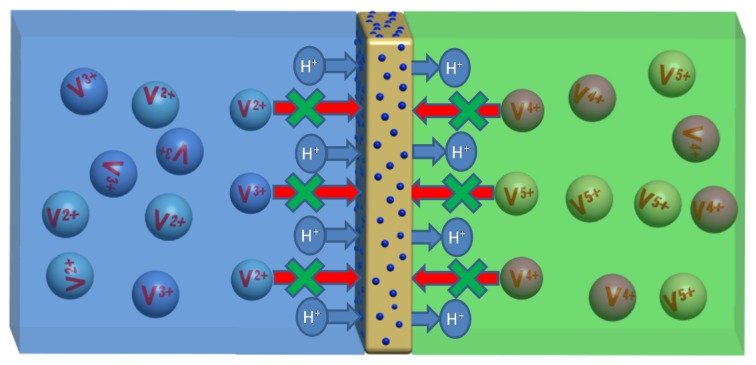
Schematic principle of the annealed composite membrane used in this study.

In this study, a self-assembly process previously developed for the preparation of polymer electrolyte membrane fuel cells was combined with an annealing method to prepare a new heat-treated Nafion/SiO_2_ composite membrane for VRB applications [23]. The self-assembled hybrid Nafion/SiO_2_ membranes were prepared via heat treatment at temperatures of 240, 270, and 300 °C. The resultant membranes were then characterized by transmission electron microscopy (TEM), small-angle X-ray scattering (SAXS), and positron annihilation lifetime spectroscopy (PALS). The primary proton conductivity and vanadium ion permeability properties of these high-temperature annealed hybrid membranes were measured and compared with those of pure Nafion membranes. Compared with the Nafion/SiO_2_ composite membrane prepared using the sol-gel process [15], the SiO_2_ nanoparticles in the composite membrane produced using the self-assembly process were more uniform in size and distribution, displaying lower vanadium ion permeability, and improved VRB performance. Higher ion selectivity between the protons and vanadium ions was exhibited due to the heat treatment process for Na-form composite membranes. Our previous studies showed that high-temperature annealing (240–270 °C) of Na-form membranes can enhance their conductivity [24]. Conductivity was increased due to the thermal changes in the cluster structure of the membrane that resulted in improved performance up to their glass transition temperatures. A representative schematic diagram of vanadium ion resistance mechanism at elevated temperatures in self-assembled Nafion/SiO_2_ composite membranes in the VRB used in this study is shown in Figure 1. A modification of the Nafion/SiO_2_ composite membrane by introducing SiO_2_ nanoparticles reduced the vanadium ion permeability without reducing the proton conductivity.

## 2. Materials and Methods

### 2.1. Preparation of Nafion/SiO_2_ Hybrid Membranes

The Nafion ionomers were purchased from Dupont Company (Wilmington, DE, USA), and the Nafion cast membrane and Nafion/SiO_2_ composite membranes were prepared as reported previously [23]. First, a certain amount of tetraethoxysilane (TEOS, Shanghai Reagent Co., Shanghai, China) was added to a Nafion/*N*-methyl-2-pyrrolidone (NMP, Shanghai Reagent Co., Shanghai, China) solution, and then the ratio of H_2_O/TEOS was adjusted to 4:1 using a HCl water solution (37 wt%) (Shanghai Reagent Co., Shanghai, China) and the mixture stirred under high speed for 8 h. Finally, Nafion ionomers with different SiO_2_ nanoparticle contents were obtained: 3%@Nafion/SiO_2_ (3 wt%), 5%@Nafion/SiO_2_ (5 wt%), 10%@Nafion/SiO_2_ (10 wt%),and 15%@Nafion/SiO_2_ (15 wt%).

### 2.2. Annealingthe Nafion/SiO_2_ Composite Membrane

The Na-form Nafion/SiO_2_compositemembranes were obtained by impregnating the as-received H-form membrane in 1 M NaOH (Shanghai Reagent Co., Shanghai, China) solution for 24 h, then washing the membranes in deionized (DI) (B1, ZhongkeWeiSi Technology Development Co., Ltd., Beijing, China) water three times. Annealing of the Na-form Nafion/SiO_2_ composition membranes was performed for 2 h at temperatures of 240, 270, and 300 °C. After annealing, all the membranes were protonized in 1 M H_2_SO_4_ solution at 80 °C for 1 h and then washed thoroughly with the DI water three times. Afterward, the membrane was washed in DI water at 80 °C for 1 h, and finally washed thoroughly with the DI water three times. The membranes were called pure Nafion, X%@Nafion/SiO_2_@240°C, X%@Nafion/SiO_2_@270°C and X%@Nafion/SiO_2_@300°C, where X = 3, 5, 10, and 15. A detailed characterization of the membranes is provided in the next section.

### 2.3. Characterization

High-resolution TEM (JEM-2100F, Japan Electronics Co., Ltd., Tokyo, Japan) was used to observe the Nafion stabilized silica nanoparticles (Nafion-SiO_2_ nanoparticles). Specimens were prepared by directly placing a drop of the solution on a thin carbon film supported by a copper mesh.

Proton conductivity of the modified membranes was measured with a self-made cell using an impedance analyzer (VersaSTAT 3, Princeton Applied Research, San Diego, CA, USA). The membrane (6 × 1 cm) was placed between two platinum (Pt) wires and fixed with Teflon plate. One Pt wire was used as the working electrode and the other as the reference and counter electrodes. Electrochemical impedance spectroscopy (EIS) (CHI604D, Chenhua Co. Ltd., Shanghai, China) was recorded in the frequency range of 10 to 100 kHz under a disturbance voltage of 10 mV at room temperature and 100% relative humidity.

The rate change of the absorbance of vanadium ions in VOSO_4_ solution was used to calculate the diffusion coefficient. The permeability of VO^2+^ through the membrane was determined according to the literature [7,25]. As shown in Figure S1a, the left side of the self-made diffusion cell was filled with 110 mL 1.0/2.5 M VOSO_4_/H_2_SO_4_ solution, and the right side was filled with 110 mL 1.0/2.5 M MgSO_4_/H_2_SO_4_ solution. Magnetic stirrers were used in both cells to prevent concentration polarization, and the effective membrane area exposed to the solution was 4.91 cm^2^. Samples from the right side were collected at regular time intervals. The VO^2+^ concentration in the sample solution was measured using an UV-2600 spectrometer (Shimadzu Co., Tokyo, Japan). The sorption spectra and the calibration curve of VOSO_4_ solution are shown in Figure S1b,c. The sample concentration was calculated by the intensity of UV-Vis curve at a wavelength of 766 nm.

The ion selectivity of the membrane (*S*) is defined as the proton conductivity (*σ*) divided by the VO^2+^ permeability (*P*), which can be calculated using the following equation [11]:

(1) $$S=\frac{\sigma }{P}$$

SAXS measurements were analyzed using a SAXSessmc^2^ (Anton Paar, Graz, Austria) equipped with a Cu anode X-ray tube (*λ*_CuK_*_α_* = 0.15418 nm). All membranes were immersed in DI water at room temperature for one day before testing.

PALS was analyzed using a fast-fast coincidence PALS (Ortec Company, Oak Ridge, TN, USA) with a time resolution of 0.230 ns for the full width at half maximum (FWHM), and 1 million counts were collected for each spectrum. A ^22^Na source was first sandwiched by approximately 20 pieces of membranes (1 × 1 cm^2^) and then covered with an Al foil. The activity of the ^22^Na source was about 10 mCi. The membranes were sandwiched in a testing chamber at room temperature and humidity. The o-Ps lifetime was analyzed and recorded as τ3.

The stress-strain behavior of the dried membranes was determined on an electronic universal testing machine (WDW-0.5 type, Shanghai Hualong, Shanghai, China) at room temperature. The membranes were cut into 40 × 5 mm samples. The samples were pulled by the machine along their longitudinal direction. The gauge length and tensile rates were 30 mm and 50 mm/min, respectively.

The chemical stability was measured by soaking the membrane into 20.0 mL of 1.0 M V(V) in 3.0 M H_2_SO_4_ solution at room temperature. The membrane was gradually oxidized by the yellow V(V) ion, leading to the formation of blue V(IV) species. The testing of VO^2+^ ion permeability was performed for soaked membranes for 21 days with a previously used method [8].

The single battery effective area was 4 cm^2^. The current was collected by two graphite polar plates and the hole components were clamped between two hardness planks. VO^2+^/VO_2_^+^/H_2_SO_4_ and V^2+^/V^3+^/H_2_SO_4_ solutions served as the positive electrolyte and negative electrolyte, respectively. The electrolytes were separately and cyclically pumped through the corresponding electrode to the battery via a magnetic drive pump with a flow rate of 48 mL/min. To limit the corrosion of the carbon felts and graphite plates, the cut-off voltage for the charge and discharge process was set to 0.8 and 1.7 V, respectively.

## 3. Results and Discussion

**Figure 2:**
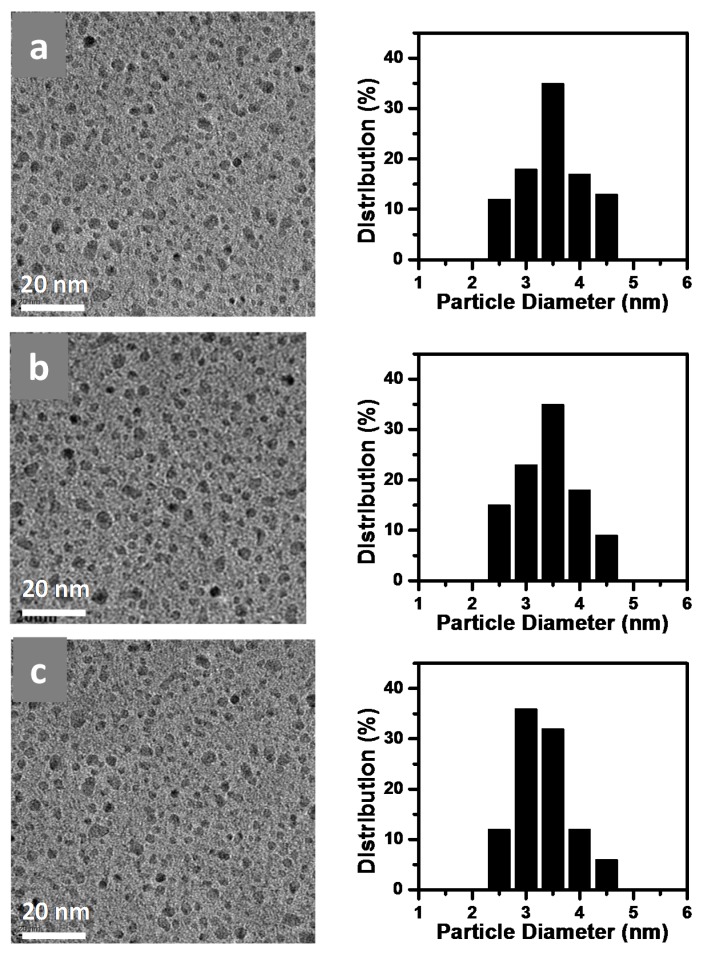
Transmission electron microscopy (TEM) micrographs and particle diameter distribution of (**a**) 5%@Nafion/SiO_2_@240°C; (**b**) 5%@Nafion/SiO_2_@270°C; and (**c**) 5%@Nafion/SiO_2_@300°C samples.

A series of annealed 5%@Nafion/SiO_2_ composite membranes annealing at 240, 270, and 300 °C were prepared and their structures analyzed via TEM (Figure 2), which revealed that the self-assembled Nafion-SiO_2_ nanoparticles were uniformly distributed. Analysis of the particle diameter distribution from TEM micrographs demonstrated that the particle diameter of 5%@Nafion/SiO_2_@240°C, 5%@Nafion/SiO_2_@270°C, and 5%@Nafion/SiO_2_@300°C samples were between 2.5 and 4.5 nm. The energy dispersive spectrometer (EDX) data of 5%@Nafion/SiO_2_@270°C sample showed that the silicon dopant had been successfully introduced into the membrane (Figure S2). Figure S3 shows the TEM micrographs and particle diameter distribution of Nafion/SiO_2_ membranes containing 3, 5, 10, and 15% Si, demonstrating that the size of SiO_2_ nanoparticles increased as the Si content increased. The particle diameters of the 3%@Nafion/SiO_2_@270°C, 5%@Nafion/SiO_2_@270°C, 10%@Nafion/SiO_2_@270°C, and 15%@Nafion/SiO_2_@270°C samples were between 1.0 and 3.5, 2.5 and 4.5, 3.5 and 7.5, and 2.5 and 7.5 nm, respectively.

**Figure 3:**
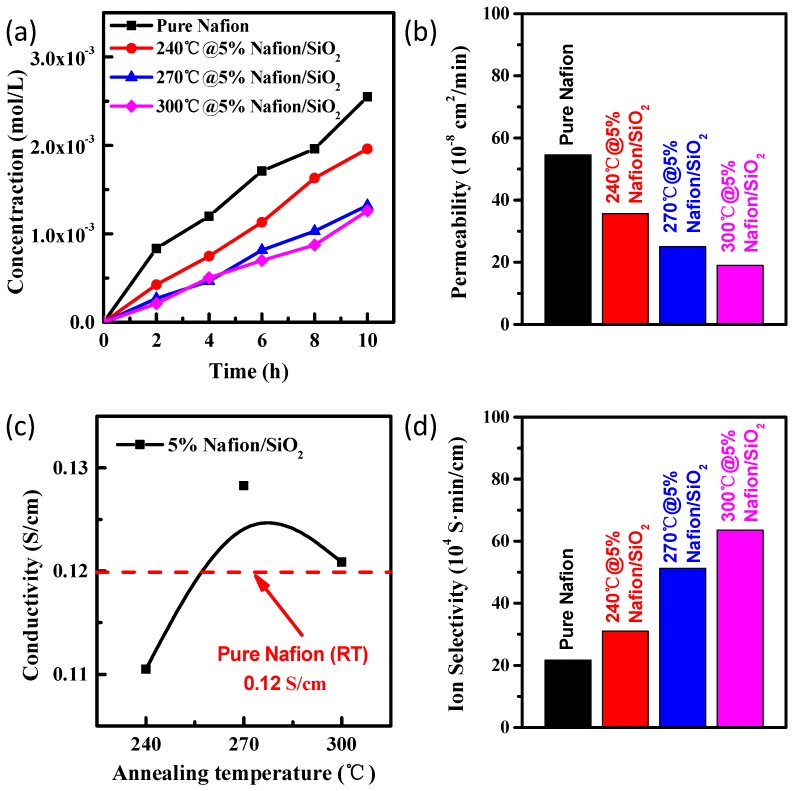
Comparison of the time dependent (**a**) vanadium ion concentrations; (**b**) permeability; (**c**) proton conductivities; and (**d**) ion selectivity of 5%@Nafion/SiO_2_@240°C, 5%@Nafion/SiO_2_@270°C, 5%@Nafion/SiO_2_@300°C and pure Nafion membranes.

The vanadium ion permeability, proton conductivity, and ion selectivity performance of all the annealed composite membranes were measured and analyzed (Figure 3 and Figure S4) which determined the effect of Si content and temperature on the ion selectivity and proton conductivity of the Nafion/SiO_2_ composite membranes. Figure 3a,b and Figure S4a,b show the results of the vanadium ion permeability of pure Nafion and Nafion/SiO_2_ composite membranes with 3, 5, 10, and 15% Si content annealing at 240, 270, and 300 °C. In VRB, the ion exchange membrane acts as an effective barrier to prevent vanadium ions from being transported between the anode to the cathode. Previous studies reported that unwanted vanadium ion crossover is a key factor affecting cell coulomb efficiency [26]. Accordingly, we decided to measure the vanadium ion permeability coefficients of both the annealed Nafion/SiO_2_ hybrid membranes and the pure Nafion membrane at room temperature (Table 1). The rate of increase in VO^2+^ concentration and the amount of vanadium ion crossover for the annealed Nafion/SiO_2_ composite membranes were significantly less compared with pure Nafion membranes. With the change in Si content, the sample with 5% Si content showed the best vanadium ion permeability (Figure S5). This may be caused by the SiO_2_ nanoparticles acting as a barrier to vanadium ion migration and/or due to the formation of smaller ion clusters at elevated temperatures, which both contribute to the formation of narrower vanadium ion channels [7,14]. In addition, thermal reorganization of the fluorocarbon backbone of the membrane may occur, allowing its hydrophobic regions to form more compacted structures at elevated temperatures, which would result in lower permeability of vanadium ions at higher temperatures [11,24]. The higher levels of Si (≥10%) in the composite membranes resulted in uneven size distribution of the SiO_2_ nanoparticles due to agglomeration (Figure S3), further affecting their vanadium ion permeability.

The proton conductivity of all the annealed composite membranes with different Si-doped content is shown in Figure 3c and Figure S4c. The results of the proton conductivity studies on pure and annealed 3%@Nafion/SiO_2_, 5%@Nafion/SiO_2_, 10%@Nafion/SiO_2_, and 15%@Nafion/SiO_2_ composite membranes at room temperature, with annealing previously completed over a temperature range of 240 to 300 °C, show that lower SiO_2_ nanoparticles contents (≤5%) only slightly impacted their performance when compared with pure Nafion. However, too much SiO_2_ nanoparticle addition (≥10%) significantly reduced the proton conductivity of the composite membrane (Figure S4c). Therefore, we only further analyzed the 5%@Nafion/SiO_2_ samples. As shown in Figure 3c, the significant influence of temperature on proton conductivity was observed for composite Nafion/SiO_2_ membranes. The proton conductivity recorded for pure Nafion, 5%@Nafion/SiO_2_@240°C, 5%@Nafion/SiO_2_@270°C, and 5%@Nafion/SiO_2_@300°C samples were 0.12 S/cm, 0.11 S/cm, 0.13 S/cm and 0.12 S/cm, respectively, which demonstrated that the proton conductivity of the composite membranes was strongly affected by the high-temperature annealing, especially at 270 °C. We propose that the annealing temperature of 270 °C results in sufficient energy being present to enable the migration of buried sulfonated groups to form cluster aggregates via the strong electrostatic interactions of the Na^+^ counterions. This thermal molecular reorganization process resulted in an increase in the membranes proton conductance [24,25,27,28,29]. However, the proton conductivity declined when the annealing temperature was higher than 270 °C, possibly because the sulfonate-group broke at higher temperatures [24].

The selectivity values (S) of these membranes can explain the balance between proton conductivity and proton selectivity. Figure 3d and Figure S4d reveal that the selectivity for proton conductivity increased for composite membranes produced at higher annealing temperatures. This may be due to a decrease in VO^2+^ permeability caused by the formation of smaller ion clusters at higher temperatures, as well as by the blocking effect of SiO_2_ particles. The proton conductivity of these annealed membranes possibly did not decrease at higher temperatures due to strong associations between their sulfonate-groups and their Na counterions, contributing to an overall increase in selectivity for proton conductance.

**Figure 4:**
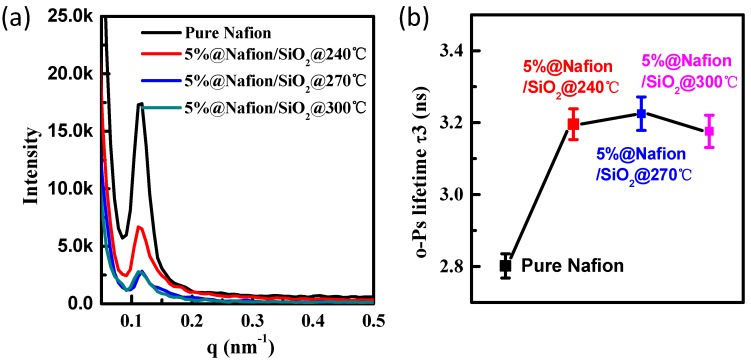
(**a**) Small-angle X-ray scattering and (**b**) positron annihilation lifetime spectroscopy of pure Nafion, 5%@Nafion/SiO_2_@240°C, 5%@Nafion/SiO_2_@270°C, and 5%@Nafion/SiO_2_@300°C membranes.

SAXS was then used to investigate whether there was a significant difference in the number of ionic domains present in the pure and annealed Nafion ionomer membranes prepared in this study (Figure 4a) [30,31]. The intensity of the SAXS patterns obtained for pure Nafion were significantly greater than those for annealed and heat-treated 5% Nafion/SiO_2_ blend membranes [32]. The peak intensities observed for the 5%@Nafion/SiO_2_@270°C and 5%@Nafion/SiO_2_@300°C membranes were almost identical, with obvious scattering peaks being observed at *q* = 0.12 that were assigned to the presence of ionic clusters in their polymeric membranes [33,34]. In addition, because the *q* values of these four membranes were almost identical, their Bragg spacing were essentially equivalent. The clear decrease in the SAXS intensity of the cluster peak for annealed 5%@Nafion/SiO_2_ membranes, when compared with pure Nafion membrane, maybe due to a difference in electron density caused by the presence of inorganic nanoparticles. The SAXS peaks for the 5%@Nafion/SiO_2_@240°C membrane were also stronger than for the 5%@Nafion/SiO_2_@270°C and 5%@Nafion/SiO_2_@300°C membranes, indicating that the number and nature of the ionic clusters present in these membranes changed when elevated temperatures were used for annealing.

PALS was then used to study the free volume of these membranes, with the o-Ps annihilation lifetime τ3 value providing a measure of their free volume element sizes and number densities [35]. Figure 4b shows the results obtained from the positron annihilation experiments of the pure Nafion, 5%@Nafion/SiO_2_@240°C, 5%@Nafion/SiO_2_@270°C, and 5%@Nafion/SiO_2_@300°C membranes. The o-Ps annihilation lifetime τ3 values for these annealed 5% Nafion/SiO_2_ composite membranes significantly increased compared to the pure Nafion membrane, with the τ3 values of the annealed membranes having a maximum value at 270 °C. This suggests that the amount of free volume in these annealed membranes increases with increasing temperature, which results in an associated increase in proton conductivity caused by the presence of a greater number of proton transporting channels at higher annealing temperatures. However, the free volume of these membranes decreased at annealing temperatures above 270 °C, which may be caused by the higher temperatures disrupting the optimal microstructure of the membrane.

**Figure 5:**
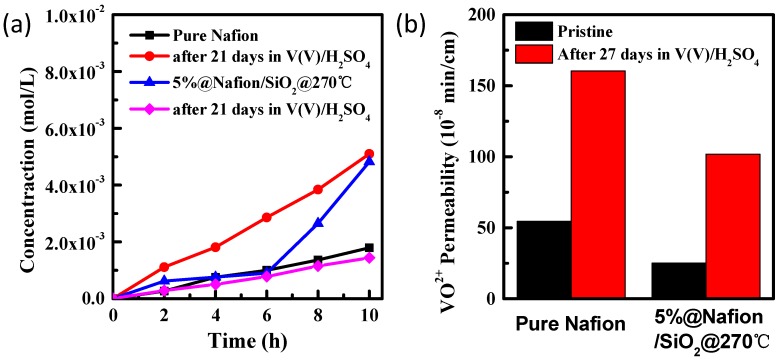
(**a**) Variation in VO^2+^ concentration over time for pure Nafion and 5%@Nafion/SiO_2_@270°C membranes before and after 21 days of immersion in V^5+^ solution; and (**b**) VO^2+^ permeability of pure Nafion and 5%@Nafion/SiO_2_@270°C membranes before and after 21 days of immersion in V^5+^ solution.

The chemical stability of the VRB membranes under highly-oxidizing conditions is critical to their life span, with poor chemical stability leading to rapid degradation of coulombic efficiency, which is often caused by the strongly-oxidizing electrolyte damaging the membrane that allows vanadium ions to permeate across the membrane [36,37]. The chemical stabilities of the pure Nafion and 5%@Nafion/SiO_2_@270°C membranes were investigated by comparing their permeabilities after they had been immersed in V^5+^ solution for 21 days (Figure 5). We found that the degree of VO^2+^ permeability for the pure Nafion membrane was higher than the Nafion/SiO_2_@270°C membrane, indicating the heat-treated annealed membrane exhibits better chemical stability than the pure Nafion membrane. The mechanical behavior of the membranes was also analyzed (Figure S6), with the Nafion/SiO_2_@270°C membrane proving to be more robust that the pure Nafion membrane. Therefore, the chemical and mechanical stability studies suggest that the Nafion/SiO_2_@270°C membrane should provide improved levels of performance for VRB applications.

**Figure 6:**
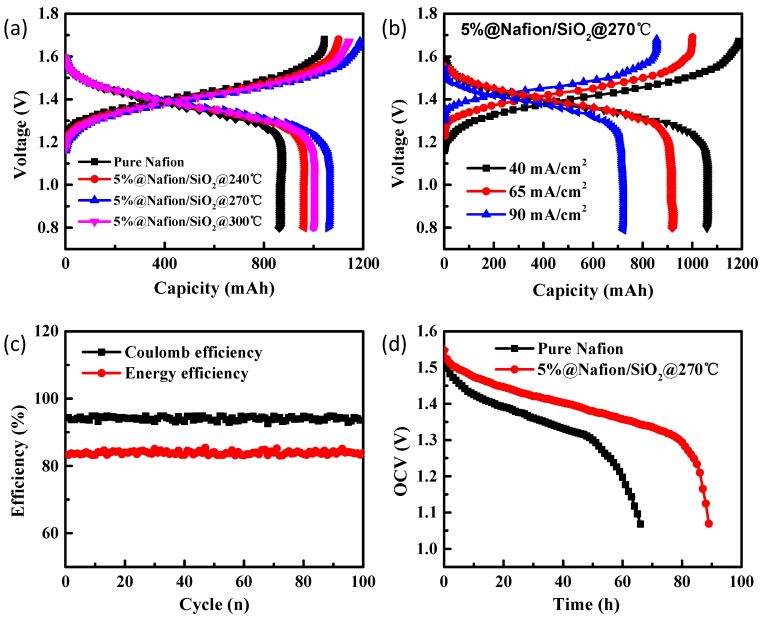
(**a**) Charge–discharge curves of vanadium redox flow batteries (VRB) containing pure Nafion, 5%@Nafion/SiO_2_@240°C, 5%@Nafion/SiO_2_@270°C, and 5%@Nafion/SiO_2_@300°C membranes at a current density of 40 mA/cm; (**b**) charge-discharge curves of VRB containing 5%@Nafion/SiO_2_@270°C membranes at different current densities; (**c**) cycle performance of VRB containing 5%@Nafion/SiO_2_@270°C membranes; and (**d**) self-discharge curves of VRFB assembled with pure Nafion and 5%@Nafion/SiO_2_@270°C membranes.

Figure 6a shows the charge-discharge curves of pure Nafion, 5%@Nafion/SiO_2_@240°C, 5%@Nafion/SiO_2_@270°C, and 5%@Nafion/SiO_2_@300°C membranes at a current density of 40 mA/cm, with heat treated membranes exhibiting higher charge and discharge capacities than the untreated Nafion membrane. The 5%@Nafion/SiO_2_@270°C membrane possessed the highest capacity and lowest charge voltage (Figure 6a), meaning that it has lower vanadium ion permeability, less overall resistance, and higher discharge voltages. These findings also indicate that the structure of the 5%@Nafion/SiO_2_@270°C membrane is optimal for preventing permeation by vanadium ions, potentially leading to improved performance and increased lifespan.

Figure 6b shows the charge-discharge plots of a single cell VRB that contains the 5%@Nafion/SiO_2_@270°C membrane at different current densities. The discharge capacity increased as the charge-discharge current density increased. This phenomenon is attributed to shorter charge-discharge time occurring at high current densities, which also minimized the number of vanadium ions passing through the membrane. The cycle performance of the VRB containing 5%@Nafion/SiO_2_@270°C membranes at a charge-discharge current of 65 mA/cm resulted in excellent coulombic efficiency (CE) and energy efficiency (EE) values of 93.5% and 83.9%, respectively (Figure 6c), meaning that almost no decay in CE and EE occurred after 100 cycles. Therefore, we concluded that annealed Nafion/SiO_2_ membranes exhibit high stability in the presence of strongly acidic electrolytes, which enables the VRB to operate continuously at a good performance level.

VRB self-discharge is caused by an increased permeability of their membranes to polar electrolytes, with the overall performance of their cells consistent with that expected of an open circuit voltage (OCV) system. The OCV of VRB containing pure Nafion and 5%@Nafion/SiO_2_@270°C membranes is shown in Figure 6d, with an initial small decrease in its OCV values being followed by a rapid decrease in potential to 1.05 V. The time required for the OCV values of VRB containing pure Nafion and 5%@Nafion/SiO_2_@270°C membranes to drop to a threshold level of 1.3 V were 68 and 90 h, respectively. This means that the rate of decay for the self-discharge of VRB containing 5%@Nafion/SiO_2_@270°C membranes was significantly lower than for a conventional VRB containing pure Nafion membrane. These results indicate that annealed Nafion/SiO_2_ membranes are less permeable to electrolyte than pure Nafion membranes, which are responsible for the performance properties for VRB applications.

## 4. Conclusions

Annealed Nafion/SiO_2_ composite membranes were prepared using a self-assembly process at elevated temperatures in the presence of alkaline metal sodium ions. These high-temperature modified composite membranes possess higher proton conductivity than conventional Nafion membranes due to an increase in the number of ion clusters and a greater free volume that resulted in an increase in the number of open proton transporting channels available for conduction. These annealed composite membranes displayed lower vanadium ion permeability than pure Nafion membranes, which is potentially due to the presence of the preferential formation of small ion clusters at higher temperatures. Higher coulombic and energy efficiencies and lower self-discharge rates were observed for single-cell VRBs containing these thermally-annealed membranes, thus indicating that their overall performance levels are better that those produced by pure Nafion membranes.

## Figures and Tables

**Table 1:** Vanadium ion permeability of pure Nafion and Nafion/SiO_2_ composite membranes.

Sample	V Ion Permeability (10^−^^8^ cm^2^/min)	Sample	V Ion Permeability (10^−^^8^ cm^2^/min)	Sample	V Ion Permeability (10^−^^8^ cm^2^/min)
Pure Nafion	54.6				
3%@Nafion/ SiO_2_@240°C	42.4	3%@Nafion/ SiO_2_@270°C	36.5	3%@Nafion/ SiO_2_@300°C	24.3
5%@Nafion/ SiO_2_@240°C	35.7	5%@Nafion/ SiO_2_@270°C	25.0	5%@Nafion/ SiO_2_@300°C	19.0
10%@Nafion/ SiO_2_@240°C	52.6	10%@Nafion/ SiO_2_@270°C	26.1	10%@Nafion/ SiO_2_@300°C	21.0
15%@Nafion/ SiO_2_@240°C	49.3	15%@Nafion/ SiO_2_@270°C	39.4	15%@Nafion/ SiO_2_@300°C	41.1

## Supplementary Materials

The following are available online at http://www.mdpi.com/2073-4360/10/5/473/s1.
