# Supplementary material for: Effect of Elevated Temperature Annealing on Nafion/SiO2 Composite Membranes for the All-Vanadium Redox Flow Battery
Source: Polymers (Basel). 2018 Apr 26;10(5):473. doi: 10.3390/polym10050473 (PMC6415359; doi:10.3390/polym10050473)
Supplement: Supplementary file 1 [file polymers-10-00473-s001.pdf]

polymers

Supplementary Materials

Effect of Elevated Temperature Annealing on Nafion/SiO2 Composite Membranes for the All-Vanadium Redox Flow Battery

Sixiu Zeng, Liuli Zeng, Rui Wang, Wei Guo * and Haolin Tang *

State Key Laboratory of Advanced Technology for Materials Synthesis and Processing, Wuhan University of Technology, Wuhan 430070, China

* Correspondence: guowei2016@whut.edu.cn (W.G.); thln@whut.edu.cn (H.T.)

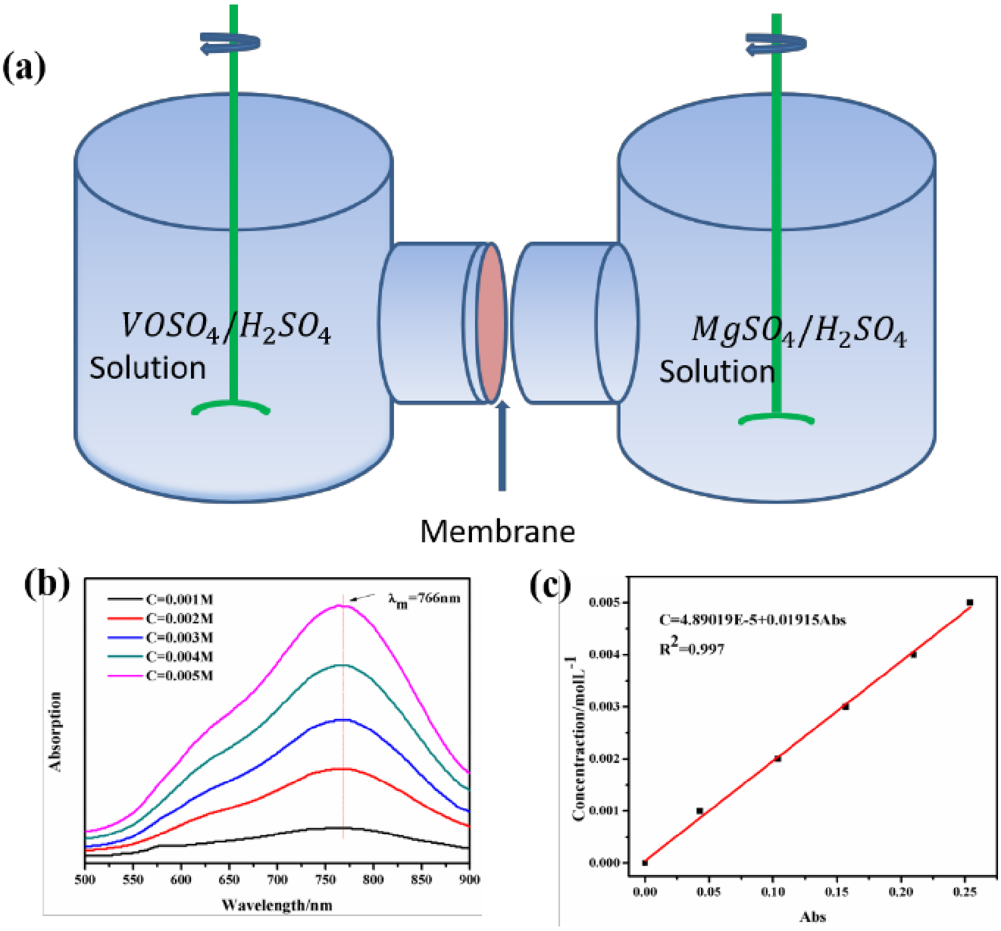

Figure S1. (**a**) Schematic diagram of the diffusion cell used to measure the VO^{2+} crossover; (**b**) sorption spectra of the $VOSO_4$ solution for four different concentrations at $\lambda_{max} = 766$ nm; and (**c**) the calibration curve of the $VOSO_4$ solution with the concentration range between 0.001 M and 0.005 M.

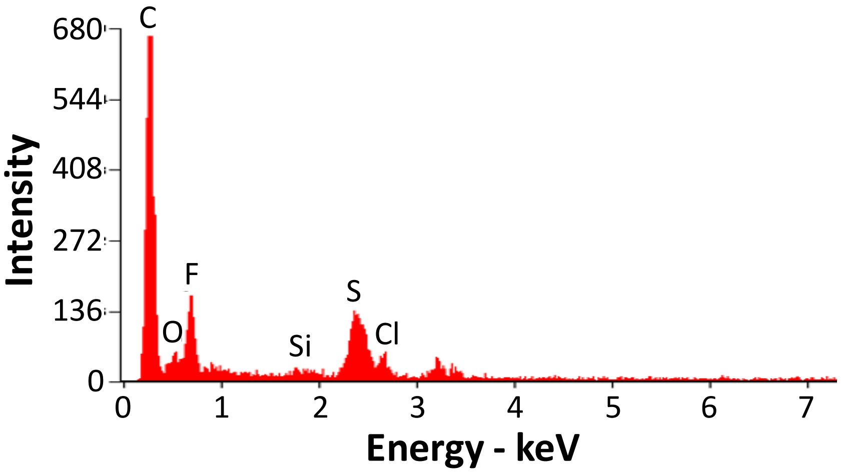

Figure S2. EDX data of the 5%@Nafion/SiO_2@270 °C sample.

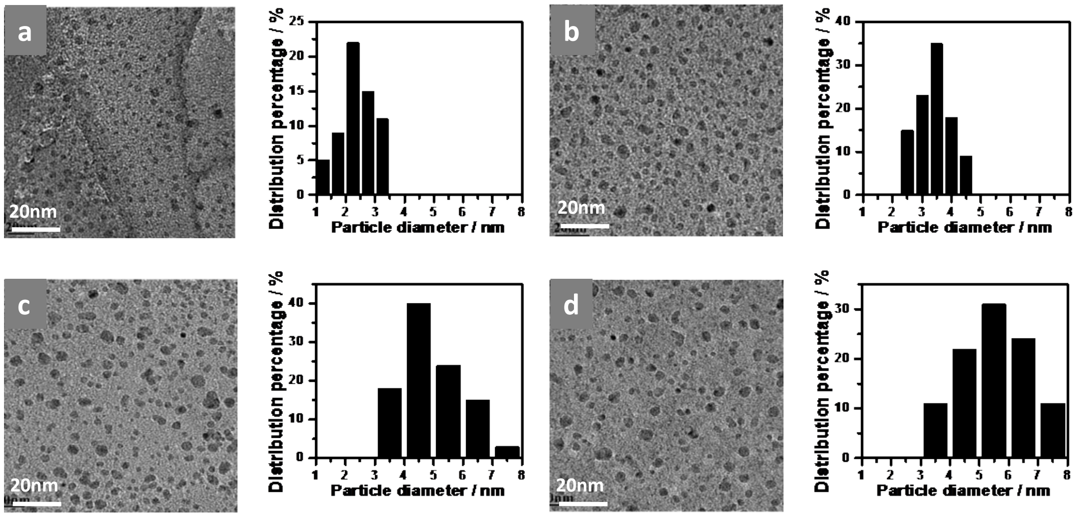

Figure S3. TEM micrographs and particle diameter distribution of 3%@Nafion/SiO_2@270 °C (**a**); 5%@Nafion/SiO_2@270 °C (**b**); 10%@Nafion/SiO_2@270 °C (**c**); and 15%@Nafion/SiO_2@270 °C (**d**) samples.

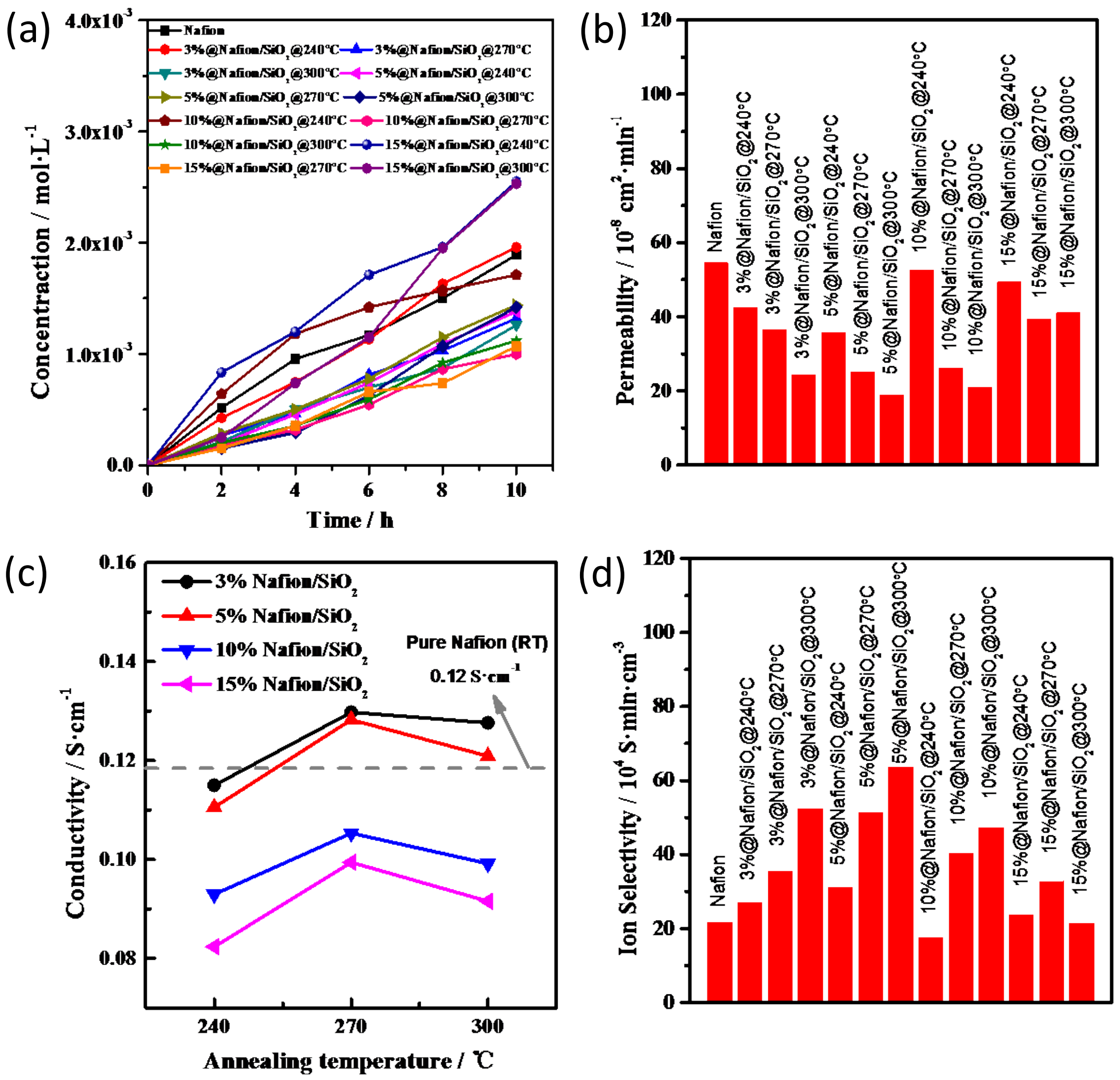

Figure S4. Comparison of the time-dependent vanadium ion concentrations (**a**), permeability (**b**), proton conductivities (**c**), and ion selectivity (**d**) of 3%@Nafion/SiO_2@240 °C, 3%@Nafion/SiO_2@270 °C, 3%@Nafion/SiO_2@300 °C, 5%@Nafion/SiO_2@240 °C, 5%@Nafion/SiO_2@270 °C, 5%@Nafion/SiO_2@300 °C, 10%@Nafion/SiO_2@240 °C, 10%@Nafion/SiO_2@270 °C, 10%@Nafion/SiO_2@300 °C, 15%@Nafion/SiO_2@240 °C, 15%@Nafion/SiO_2@270 °C, 15%@Nafion/SiO_2@300 °C, and pure Nafion samples.

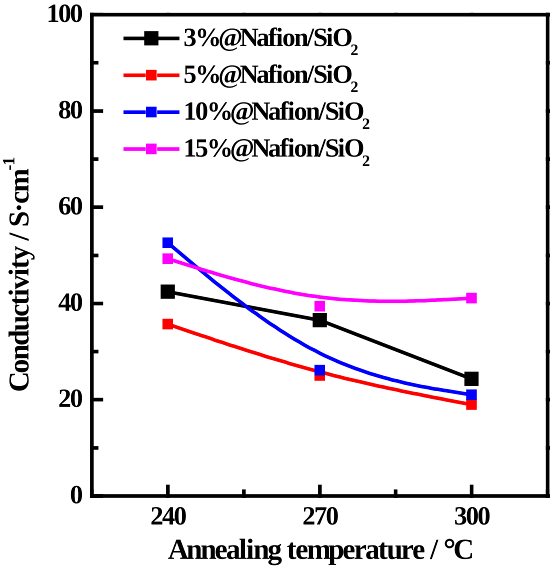

Figure S5. The ratio of proton conductivity and temperature of 3%@Nafion/SiO_2, 5%@Nafion/SiO_2, 10%@Nafion/SiO_2, and 15%@Nafion/SiO_2 samples.

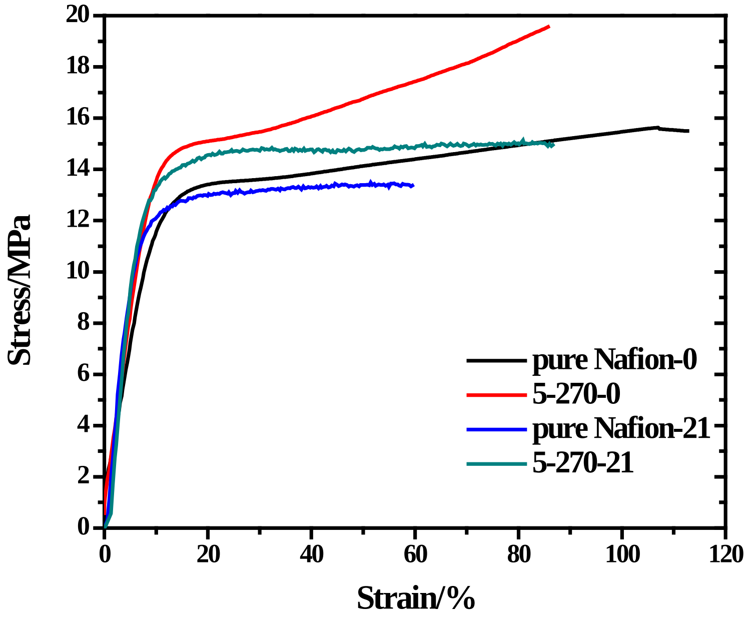

Figure S6. The stress-strain behavior for pure Nafion and 5%@Nafion/SiO_2@270 °C samples before and after 21 days of immersion in V^{5+} solution.

© 2018 by the authors. Submitted for possible open access publication under the terms and conditions of the Creative Commons Attribution (CC BY) license (http://creativecommons.org/licenses/by/4.0/).
